# Rhythm perception and production predict reading abilities in developmental dyslexia

**DOI:** 10.3389/fnhum.2014.00392

**Published:** 2014-06-04

**Authors:** Elena Flaugnacco, Luisa Lopez, Chiara Terribili, Stefania Zoia, Sonia Buda, Sara Tilli, Lorenzo Monasta, Marcella Montico, Alessandra Sila, Luca Ronfani, Daniele Schön

**Affiliations:** ^1^Child Neurology and Psychiatry Ward, Institute for Maternal and Child Health - IRCCS Burlo Garofolo Pediatric InstituteTrieste, Italy; ^2^Center for the Child Health – OnlusTrieste, Italy; ^3^Developmental Neuropsychiatry Ward, Villaggio Eugenio LittaRome, Italy; ^4^Epidemiology and Biostatistics Unit, Institute for Maternal and Child Health - IRCCS Burlo Garofolo Pediatric InstituteTrieste, Italy; ^5^Institut de Neurosciences des Systémes, Aix-Marseille UniversitéMarseille, France; ^6^INSERM, U1106Marseille, France

**Keywords:** dyslexia, phonological awareness, temporal processing, rhythm, music

## Abstract

Rhythm organizes events in time and plays a major role in music, but also in the phonology and prosody of a language. Interestingly, children with developmental dyslexia—a learning disability that affects reading acquisition despite normal intelligence and adequate education—have a poor rhythmic perception. It has been suggested that an accurate perception of rhythmical/metrical structure, that requires accurate perception of rise time, may be critical for phonological development and subsequent literacy. This hypothesis is mostly based on results showing a high degree of correlation between phonological awareness and metrical skills, using a very specific metrical task. We present new findings from the analysis of a sample of 48 children with a diagnosis of dyslexia, without comorbidities. These children were assessed with neuropsychological tests, as well as specifically-devised psychoacoustic and musical tasks mostly testing temporal abilities. Associations were tested by multivariate analyses including data mining strategies, correlations and most importantly logistic regressions to understand to what extent the different auditory and musical skills can be a robust predictor of reading and phonological skills. Results show a strong link between several temporal skills and phonological and reading abilities. These findings are discussed in the framework of the neuroscience literature comparing music and language processing, with a particular interest in the links between rhythm processing in music and language.

## Introduction

Music is a complex activity that taps onto several sensory-motor, cognitive and emotional mechanisms. Over the last two decades many studies have tested the hypothesis that music training (implying formal training and/or regular practice) can impact non-musical abilities. Most of these studies have addressed this issue by comparing a population of musicians, either professional or amateur, and a population of non-musicians, namely participants with little or no music training. Overall, these studies have shown a clear effect of music-dependent brain plasticity affecting brain activity both at the functional and structural level in adults (Herholz and Zatorre, 2012) and children with as little as one year of musical practice (Hyde et al., 2009).

Music shares many basic processes with other human activities, and this is particularly evident when comparing music and speech (Besson and Schön, 2011). Both rely on sound processing and require a precise—though often categorical—representation of several sound features, such as timbre, pitch, duration, and their interactions. As an example, these representations allow discrimination between *legato* and *staccato* violin sounds as well as [ba] and [pa] phonemes.

While a common belief is that music is mostly challenging with respect to pitch, music making puts a high challenge on all these sound features, and most importantly on complex spectral features, because sound quality (and not just being in tune) is what a musician has to work on from the very start. This may explain why music training enhances processing of sound features that play a major role in speech processing as well (Kraus and Chandrasekaran, 2010). Adult musicians have a more faithful representation of speech sound features in the brainstem, both in terms of pitch and formants (Wong et al., 2007). These representations are also more robust to noisy conditions (Parbery-Clark et al., 2012). This subcortical music-induced plasticity may depend upon the numerous corticofugal (descending) projections from the cortex to the brainstem auditory relays.

One of the most important properties of music being its structuring sounds in time and in a tonal space, it is not surprising that music-dependent brain plasticity goes well beyond subcortical and primary auditory and sensorimotor cortex, thus affecting more integrated functions. For instance, there is evidence that music training facilitates language learning. Children taking music classes are better at segmenting a new artificial language on the sole basis of its statistical properties (François et al., 2012), an ability that seems to rely heavily on the dorsal pathway (Rodriguez-Fornells et al., 2009). Other studies show an overall enhancement of verbal intelligence in children taking music classes (Moreno et al., 2011), possibly tapping onto several integrated brain functions.

A number of studies have also reported an association between music and reading skills. For example, pitch perception was positively correlated with phonemic awareness and reading abilities in children (Anvari et al., 2002) and the variability in tapping to a beat correlated with performance on reading and attention tests (Tiernay and Kraus, 2013a). A meta-analysis of 25 cross-sectional studies found a significant association between music training and reading skills (Butzlaff, 2000). Importantly, music seems to be able, to a certain extent, to drive an improvement in reading skills in normal readers (Moreno et al., 2009).

The fact of showing, on one side that music and language share several sensory and cognitive processes, and on the other side that music training enhances several language abilities, has brought several researchers to hypothesize that music training may be effective in rehabilitation of several motor and cognitive disorders in different clinical populations (Tallal and Gaab, 2006; Besson et al., 2007; Särkämö et al., 2008; Schön et al., 2008; Altenmüller et al., 2009; Kraus and Chandrasekaran, 2010; Goswami, 2011; Patel, 2011; Amengual et al., 2013).

Our study focuses on the relation between musical abilities and reading skills in children with developmental dyslexia. Developmental dyslexia is a disorder characterized by a specific and long lasting difficulty in reading acquisition, limited to written text decoding with no sensory or neurological deficits (Snowling and Hulme, 2012).

Reading results are slow and inaccurate, despite adequate intelligence, socio-cultural background and instruction. Difficulties arise typically from a phonological core deficit with an indirect impact on reading comprehension, requiring lexical, morpho-syntactic, memory and prediction abilities that are not directly affected by this disorder (Lyon et al., 2003).

In Italy, prevalence of developmental dyslexia ranges from 1.5 to 5% (Cornoldi and Tressoldi, 2007). A recent epidemiological study involved a sample of more than 1500 children attending the fourth grade of primary school in Friuli Venezia Giulia, a region in the north of Italy, and found prevalence slightly higher than 3%, thus lower than that reported in opaque language speaking countries such as United Kingdom or France (Barbiero et al., 2012).

While the neurobiological and genetic basis of developmental dyslexia is now widely accepted in the scientific community, it is not clear whether there is a specific neuropsychological function that, once impaired, determines such heterogeneous landscape of difficulties in reading acquisition. Indeed, if the reading disorder is best described in terms of phonological deficits and to a certain extent visual deficits, there are other deficits of working memory, sequencing, mental calculation, motor coordination or music processing that are often associated with the main reading disorder (Ramus, 2004; Snowling and Hulme, 2012).

These observations have brought to the emergence of multiple hypotheses relative to the functional deficit of developmental dyslexia that may be accounted for by a multifocal brain abnormality approach (Pernet et al., 2009). Nonetheless, several authors agree in defining the phonological deficit as the core deficit of developmental dyslexia, primarily due to a dysfunction of the auditory system yielding a poor temporal processing. Interestingly, several studies have shown that children with developmental dyslexia also show an impairment of music temporal processing; compared to normally developing children they are impaired in tapping along a song (Overy et al., 2003), show greater variability when asked to tap along a metronome (Thomson and Goswami, 2008) and are quite poor in segmentation and grouping tasks, both in speech and music (Petkov et al., 2005). Furthermore, Wolff (2002) found that children with dyslexia tended to overanticipate the cued stimulus by as much as 100 ms, unlike their control matched peers, and showed difficulties reproducing patterned rhythms of tones.

What still remains to be understood is the precise temporal scale(s) that may be impaired, thus causing a phonological deficit. For instance, Tallal (1980, 2004) has suggested a rapid temporal processing deficit which would prevent the discrimination of different phonemes, in particular contrastive consonants such as [t]-[d] that acoustically differ in terms of rapid transient formants. While several studies supported a notion of causal link between impaired perception of rapid spectrotemporal cues and impaired literacy (Reed, 1989; De Martino et al., 2001; Tallal, 2004), recent research has suggested a rather limited role for rapid auditory processing in developmental dyslexia (Heath and Hogben, 2004a,b).

An alternative hypothesis seems to rely on a longer time scale, that of amplitude envelope, and more precisely that of “rise time” which in the case of speech can be very important to distinguish different voice onset times (VOT) allowing to categorize /ch/ of chip vs. /sh/ of ship or /b/ of bull vs. /p/ of pool (Rosen, 1992). There is, indeed, growing literature attesting the presence of impaired amplitude envelope perception in developmental dyslexia, across languages with different phonological structures and languages with different writing systems (for a review see Goswami et al., 2011b, 2013). More precisely, a specific deficit in accurately processing sound rise time (the time taken for sounds to reach their maximum amplitude) has been postulated (Goswami et al., 2010). Rise times are critical in speech signal, as they reflect the patterns of amplitude modulation that facilitate syllabic segmentation. Thus, a poor perception of amplitude envelope structure may lead to poor phonological development (Goswami, 2011). By contrast to rapid spectrotemporal modulations, more linked to acoustic processing, slower spectrotemporal modulations and the amplitude envelope are linked to syllabic and prosodic structure, in particular to speech rhythm and intonational patterning (Greenberg, 2006).

Impaired auditory perception of slow (<10 Hz) temporal modulations in speech is thus likely to cause poor perception of speech rhythm and syllable stress (Goswami, 2011; Leong et al., 2011). Indeed, children with developmental dyslexia have a deficit in both rhythm and meter perception, also when using musical stimuli (Huss et al., 2011).

Following the idea of a neural oscillatory phase-locking to speech modulation patterns (e.g., Ghitza, 2011; Giraud and Poeppel, 2012), the perceptual difficulties commonly observed in developmental dyslexia could be underpinned by impaired phase alignment between speech and neural activity as well as poor firing coupling between different neuronal oscillatory rates (Abrams et al., 2009; Lehongre et al., 2011; Leong and Goswami, 2014).

In this work we present data collected on an Italian highly selected sample of children with developmental dyslexia. In the light of what has been documented in the literature, we investigate the relation between musical temporal, phonological, and decoding (reading) skills. The starting point is the hypothesis of a temporal sampling deficit as possible cause of the poor phonological representation and reading ability. We present a multivariate approach first describing correlations between reading and temporal processing outcomes. Then, we analyse, within the limits of a cross sectional approach, the (predictive) links between several “temporal processing” measures and reading abilities. Finally, we interpret our findings within the theoretical framework described above and give our contribution to the development of a targeted and rehabilitative hypothesis of developmental dyslexia via music training.

## Methods

### Participants

Out of 225 children aged 8–11 years with a diagnosis of developmental dyslexia, referred to the health units and rehabilitation centers (IRCCS Burlo Garofolo and ASS1 local health units in Trieste and Villaggio Eugenio Litta in Grottaferrata, Rome), we included 48 children based on the following criteria.

#### Inclusion criteria

Italian native language; reading performance (accuracy and/or speed) failed on at least two of three school grade standardized Italian tests, as stated in the Original National Guidelines (PARCC DSA, 2011): text, words, pseudowords (speed scores: *z*-score <-1.8 standard deviations from the mean, accuracy: <5th percentile); hearing, vision and neurological examination within normal range; normal or corrected-to-normal visual acuity; General IQ >85 at the Wechsler Intelligence Scale for Children III.

#### Exclusion criteria

Comorbidity with Attentional Deficit Disorders with Hyperactivity (ADHD), Specific Language Impairment (SLI), Oppositional Defiant Disorder (ODD), severe emotional-relational impairments, previous formal musical or painting education for more than one year, on-going treatment.

The assessment was carried out by neuropsychologists and neurologists. Children participated only upon formal signed informed consent from their parents.

After the enrolment, the 48 children underwent the following neuropsychological assessment, which includes standardized test and phonological and musical tasks (22 children in Trieste and 26 in Grottaferrata), with mean age of 9 years and 8 months. Two children did not complete the testing.

### Neuropsychological assessment

Parents completed a detailed anamnestic questionnaire providing information about their child's health, relevant family history, and socioeconomic background.

### Standardized ability tests

#### General cognitive abilities

General cognitive abilities and working memory were assessed using the Wechsler Intelligence Scale for Children III (Orsini and Picone, 2006).

#### Auditory attention

Auditory Attention was measured using a subtest from the BIA Battery (Marzocchi, 2010) wherein children have to count the number of occurrences of a given sound.

#### Phonological awareness

Phonological awareness was assessed using the pseudowords repetition test from the Promea Battery (Vicari, 2007).

#### Reading abilities

The ability to read a text aloud was measured using an Italian standardized test for reading abilities (*MT Reading test*, Cornoldi and Colpo, 2011). Because different texts were used depending upon the school grade, statistics were based on the standardized clinical cut-off.

The ability to read single words and pseudowords aloud was measured on a standardized list of 102 Italian words and 48 Italian pseudowords (*DDE-2*, Sartori et al., 2007). Again, statistics were based on the standardized clinical cut-off (percentiles).

### Phonological awareness tasks

#### Phonemic blending

The phonemic blending test included 38 words (nouns) of increasing difficulty, selected from VARLESS Italian data base (Burani et al., 2011). Difficulty was estimated on the basis of the number of syllables, frequency in oral speech and written language, accent regularity, and orthographic complexity. Children had to blend sounds into words (e.g., hear [d]-[o]-[g] and produce [dog]). Every child performance was recorded with the Open Source sound editor and recorder Audacity 1.3 (beta). Dependent variables: number of correct items and time to perform the test.

#### Phonemic segmentation

The phonemic segmentation task also included 38 words, with the same selection criteria described above for the phonemic blending task. Children had to segment every word into its basic sounds (e.g., hear [frog] and produce [f]-[r]-[o]-[g]). Every child performance was recorded with Audacity 1.3 (beta). Dependent variables: number of correct items and time to perform the test.

### Psychoacoustic tasks

#### MLP amplitude envelop onset (rise time)

In this experiment children listened to a sequence of three identical pure tones (800 ms each) with headphones. The onset of one of the tones was varied adaptively (longer ramping) to find the subject's threshold using a Maximum Likelihood Procedure (MLP, Grassi and Soranzo, 2009). Children had to detect the longest ring tone (first, second or third?) by choosing one of three telephone pictures.

#### MLP temporal anisochrony

In this experiment children listened to a sequence of five identical complex tones (100ms each) with headphones and had to report whether or not a cartoon rabbit was able to perform regular jumps. The gap between tones 3–4 and 4–5 was varied adaptively to find the subject's threshold using a Maximum Likelihood Procedure (MLP, Grassi and Soranzo, 2009).

### Musical tasks

#### Tapping

Children had to tap along a 90 pulse/minute metronome for 40 s. Each sound lasted 50 ms, was built using a sinusoidal sound (*f* = 1200), and ramped with a 1 ms ramp at the onset and offset. Children listened to the metronome using an open headphone at approximately 75 dB and performed the task holding a pencil in their dominant hand and tapping it on a wooden box containing a microphone. They were instructed to tap as regularly as possible and did a short training before the recording to verify that they understood the task. Stimulation and acquisition were run using Audacity 1.3. Tap onsets were calculated using a custom Matlab program and a semi-automatic (supervised) procedure. Analyses were run on the coefficient of variation (i.e., the mean of the inter-tapping intervals divided by the standard deviation).

#### Rhythm reproduction

Children had to listen and reproduce 10 different rhythms (3–8 tones each; durations spanned from triplets of eight notes to half notes). Each sound of the sequence lasted 65 ms and was built using a MIDI woodblock sound. The sequences were taken and adapted from Fries and Swihart study (1990). Children listened to the sequence using an open headphone at approximately 75 dB and immediately reproduced it holding a pencil in their dominant hand and tapping it on a wooden box containing a microphone. They were instructed to tap as accurately as possible and did a short training before the recording to verify that they understood the task. Stimulation and acquisition were run using Audacity 1.3.

Every item performance was scored by two independent judges from 1 to 9 depending on its similarity to the template stimulus (9 = identical). The final mark for each child was the average of the twenty scores (inter judge correlation was 0.89).

#### Perception of musical meter

The musical meter task tested and published by Huss et al. (2011) was adapted for this study. Only trials that had metrical structure critical for children with developmental dyslexia were selected. Therefore the task included 18 trials of different metrical arrangements of a series of notes with an underlying pulse rate of 500 ms (120 bpm), each series being delivered twice within one trial. Half of the trials delivered an identical series of notes twice (“same” trials), and half delivered two slightly different series of notes (“different” trials). In the “different” trials, the change in metrical structure was caused by adding 100 ms to the accented notes. The task was to make a same-different judgment. Same and different trials were delivered in pseudo-random order.

Each sequence comprised a simple rhythm (2–5 notes) repeated 3 times, to keep short-term memory demands low. Trial length was approximately equated across variations in the number of notes by varying the length of individual notes. Ten trials (5 same, 5 different) were in 4/4 time and 8 trials (4 same, 4 different) were in 3/4 time, with accent conveyed by increasing the intensity of the relevant note in the sequence by 5 dB.

### Statistical analysis

Statistical analysis was performed with SPSS 13.0 and Intercooled Stata 9.0.

Spearman correlation analysis (based on ranks) was performed to test the strength of a relationship between variables. The 95% confidence interval for Rho was calculated with Fisher method.

The interdependence among the measured variables, namely the joint measured variations in response to possible latent (unobserved) variables, was calculated by using a factor analysis with Varimax rotation (maximizing the variances of the squared correlations between variables and factors).

Logistic regression analyses were carried out in order to evaluate which measures were associated with the six dependent variables of the reading tests. All associations were adjusted for sex, school level, city of recruitment and IQ were always controlled (see Tables 7, 8). Reading outcomes were dichotomized into highly pathological and pathological to increase robustness of the test.

## Results

**Figure 1:**
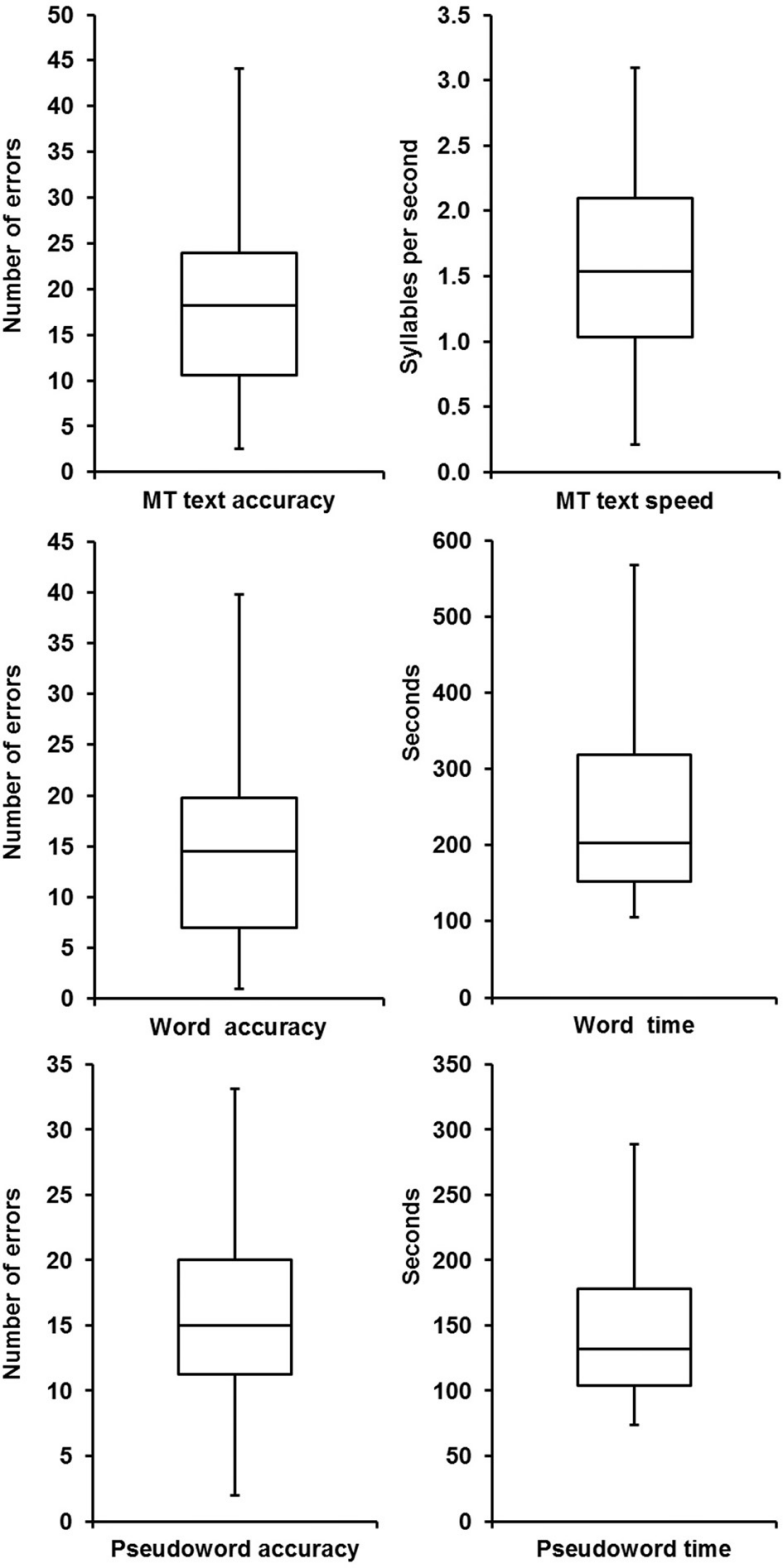
**Box plots of the reading outcomes**. The bottom and top of the box show the first and third quartiles, the band inside the box the median. The edges of the whiskers represent the values closest to the median between the minimum absolute value and Q1-1.5IQR for the lower whisker, and the maximum absolute value and Q3+1.5IQR for the upper whisker, where Q1 and Q3 are the first and third quartiles respectively, and IQR is the interquartile range.

**Figure 2:**
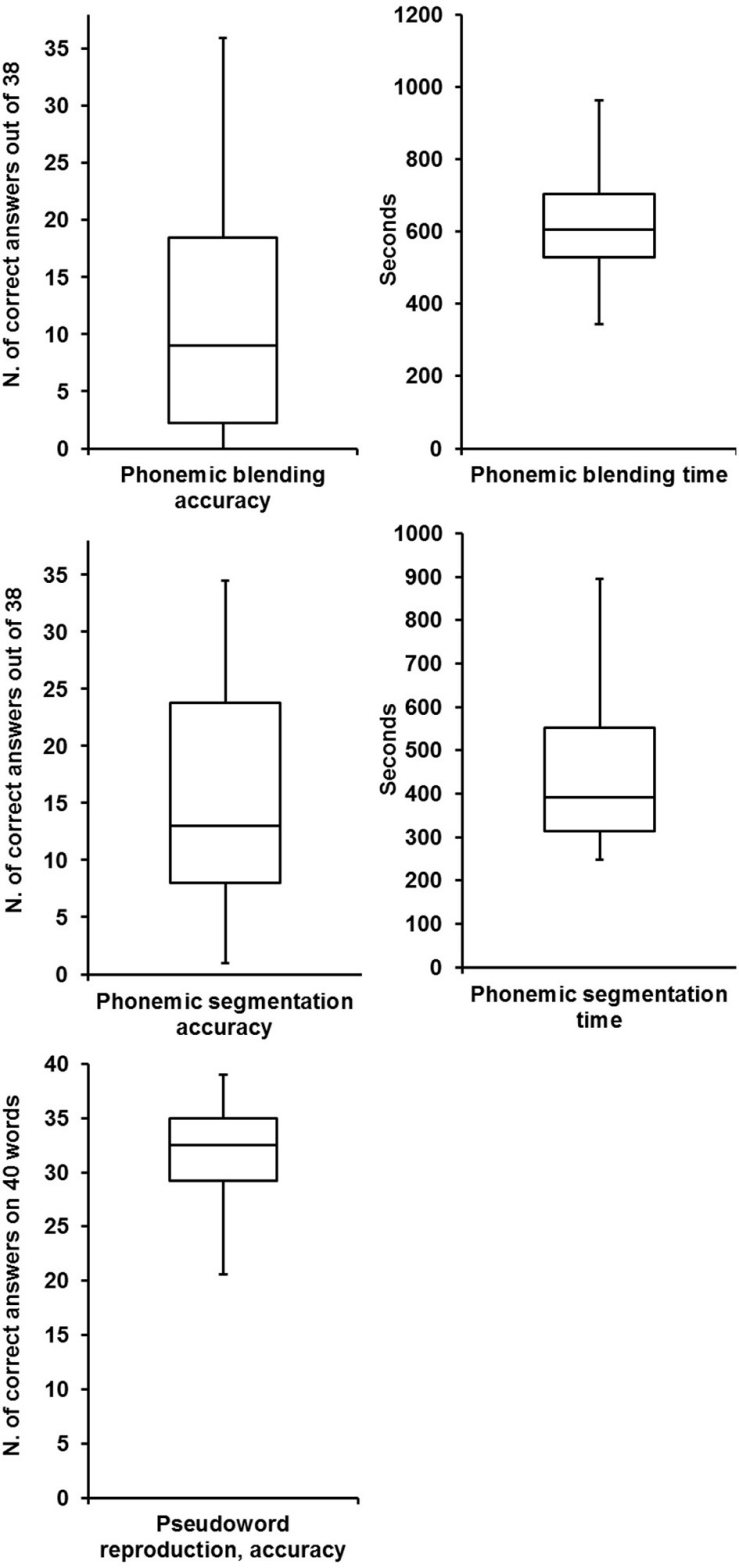
**Box plots of the phonological awareness measures**.

**Figure 3:**
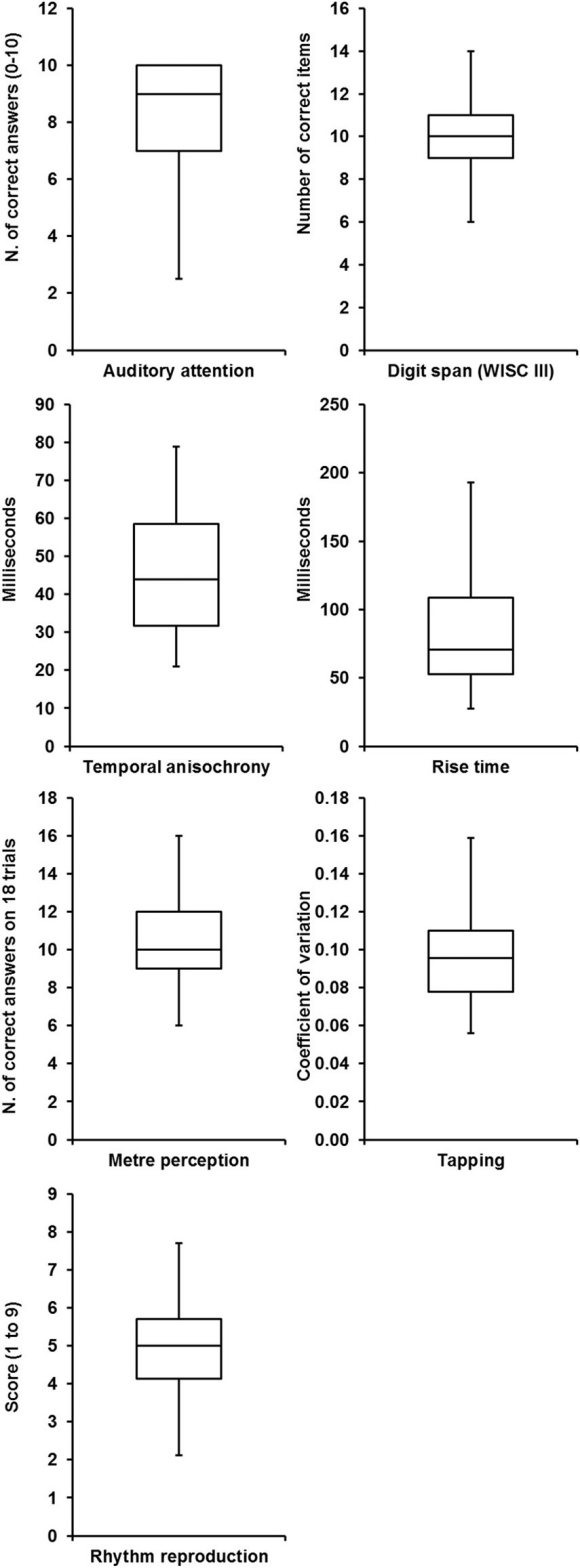
**Box plots of the temporal processing measures**.

Figures 1–3 illustrate the outcomes of reading, phonological awareness and temporal processing tests.

### Correlations

Correlations between all the temporal processing tasks and measures of phonology and literacy are provided in Tables 1, 2. An overview of significant values in Table 1 (^**^p < 0.001 and ^*^p < 0.05) shows that each reading outcome measure, with the exception of the MT text reading test, correlated significantly with rhythm reproduction and tapping tasks. The difference observed for the MT test may be due to the fact that it includes different school-level adapted texts, which in turn increases variability. Nevertheless, the outcome of this test correlates with amplitude envelope onset (rise time). Perception of the musical meter task shows a weak correlation with word reading time measure but a strong correlation with auditory attention test (*r* = 0.434, *p* < 0.01). The auditory attention test also correlates with WISC III digit span test (*r* = 0.378, *p* < 0.01) and rhythm reproduction (*r* = 0.292, *p* < 0.05), but not with phonological awareness or other reading outcomes.

**Table 1:** **Spearman correlations between reading measures and phonology and temporal processing tasks**.

	**Tapping**	**Rhythm reprod**.	**Meter percep**.	**Rise time**	**Temporal anisoch**.	**Pseudo-word reproduct. accuracy**	**Phonemic segment. accuracy**	**Phonemic blending accuracy**
MT text accuracy	0.006 (−0.288/0.299)	−0.280 (−0.528/0.011)	−0.154 (−0.425/0.143)	0.301 (0.012/0.544)	−0.135 (−0.409/0.162)	−0.165 (−0.434/0.132)	−0.342 (−0.575/−0.057)	−0.131 (−0.406/0.166)
MT text speed	−0.346 (−0.578/−0.062)	0.387 (0.109/0.609)	0.269 (−0.023/0.519)	−0.274 (−0.523/0.018)	−0.161 (−0.431/0.135)	0.240 (−0.054/0.495)	0.065 (−0.229/0.349)	0.305 (0.0159/0.547)
Word accuracy	**0.360** (0.074/0.591)	**−0.445** (−0.651/−0.178)	−0.245 (−0.499/0.049)	0.204 (−0.091/0.467)	0.278 (−0.013/0.526)	−0.224 (−0.483/0.071)	−0.168 (−0.437/0.128)	**−0.380** (−0.603/−0.101)
Word time	**0.452** (0.182/0.658)	**−0.438** (−0.646/−0.170)	−0.317 (−0.556/−0.029)	0.239 (−0.055/0.495)	0.189 (−0.108/0.454)	−0.238 (−0.495/0.056)	−0.025 (−0.313/0.267)	**−0.396** (−0.615/−0.119)
Pseudoword accuracy	0.191 (−0.108/0.459)	**−0.357** (−0.586/−0.074)	−0.162 (−0.432/0.134)	0.303 (0.014/0.545)	0.000 (−0.290/0.291)	−0.285 (−0.531/0.006)	−0.189 (−0.454/0.107)	−0.170 (−0.439/0.126)
Pseudoword time	0.292 (−0.001/0.539)	−0.229 (−0.487/0.065)	−0.284 (−0.530/0.007)	0.069 (−0.226/0.352)	0.159 (−0.138/0.429)	−0.123 (−0.399/0.174)	−0.020 (−0.308/0.272)	0.312 (-0.553/−0.024)

*In parenthesis the 95% confidence interval. Critical values (double-tailed) levels of significance for our sample size are 0.294 and 0.347 for p values of 0.05 and 0.01 respectively (not corrected for multiple comparisons) and 0.472 for p = 0.05 Bonferroni corrected for multiple comparisons. Values with a non-corrected p value < 0.01 (reasonably controlling for false positive) are reported in bold*.

**Table 2:** **Spearman correlations between temporal processing tasks and phonology tasks**.

	**Tapping**	**Rhythm reproduction**	**Meter perception**	**Rise time**	**Temporal anisochrony**
Pseudoword reproduction, accuracy	**−0.380** (−0.606/−0.097)	**0.380** (0.100/0.603)	0.131 (−0.165/0.406)	−0.209 (−0.470/0.087)	−0.246 (−0.500/0.048)
Phonemic segmentation accuracy	−0.252 (−0.508/0.045)	0.340 (0.055/0.574)	0.200 (−0.095/0.464)	−0.090 (−0.371/0.205)	−0.015 (−0.304/0.277)
Phonemic blending accuracy	**−0.527** (−0.710/−0.276)	**0.442** (0.173/0.649)	0.259 (−0.034/0.511)	−0.015 (−0.304/0.277)	−0.101 (−0.380/0.195)

*Critical p values are the same as in Table 1 exception made for the Bonferroni corrected p value, here 0.428. Values with a non-corrected p value < 0.01 (reasonably controlling for false positive) are reported in bold*.

As observed in Table 2, rhythm reproduction and tapping measures correlate with phonological tests, in particular with phonemic blending task and pseudoword repetition tests.

**Figure 4:**
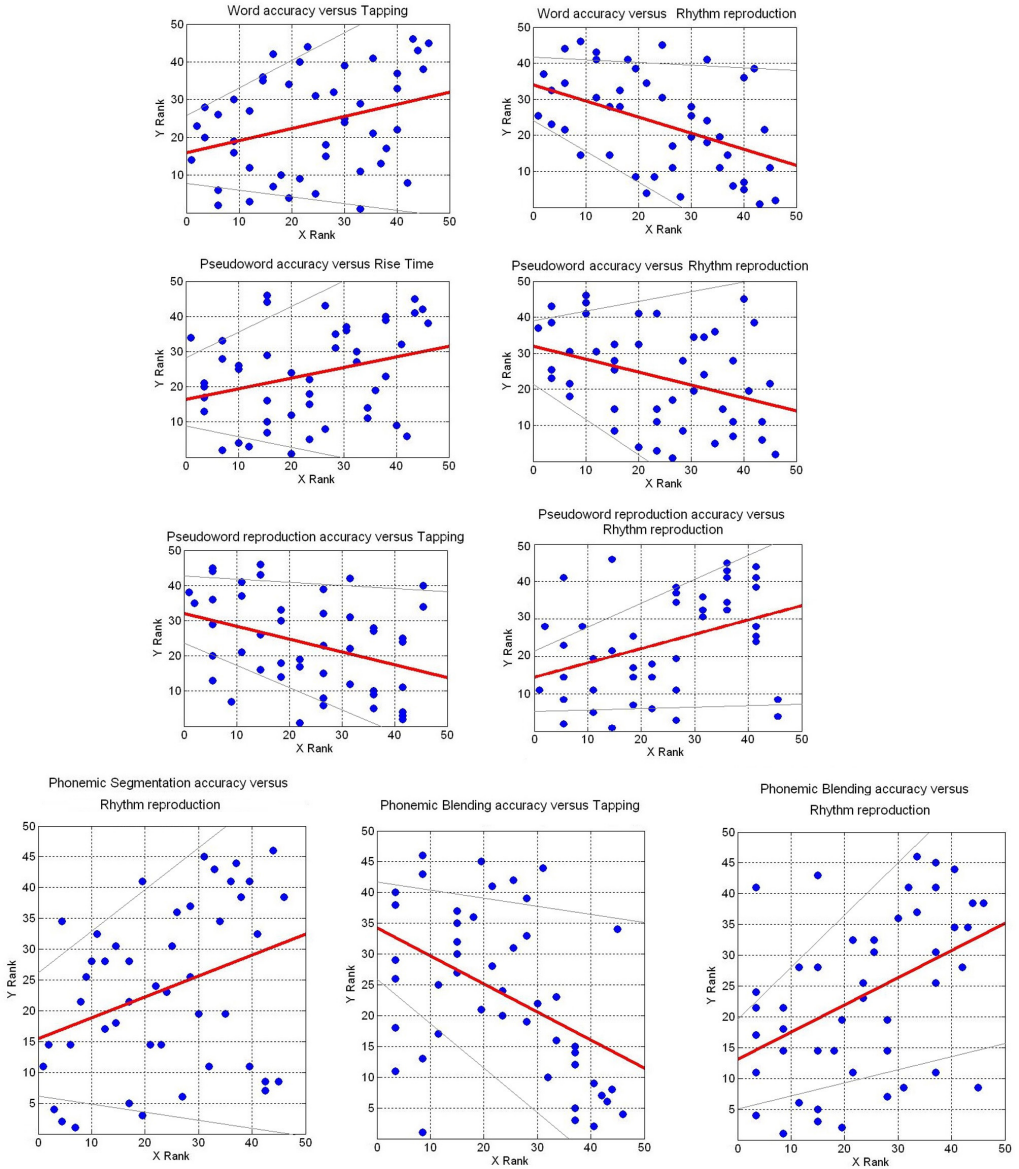
**Scatter plots of ranked variables to illustrate high *r* values between temporal tasks and reading and phonological tasks**. Red lines indicate the linear regression. Gray lines indicate 98.5 confidence interval.

Overall, Tables 1, 2 suggest that there is a strong relationship between reading outcomes, phonological awareness, and rhythm reproduction and tapping measures (Figure 4). The interdependence among these variables was tested with a factor analyses.

Table 3 shows the correlation between the different temporal tasks. Overall and as expected there is a rather strong correlation between tasks, exception made for the task measuring the rise time threshold which only shows a weak to moderate correlation with the meter perception task.

**Table 3:** **Spearman correlations between temporal processing tasks**.

	**Tapping**	**Rhythm reproduction**	**Meter Perception**	**MLP Rise time**	**MLP Temporal anisochrony**
Tapping					
Rhythm reproduction	**−0.618** (−0.771/−0.396)				
Meter Perception	**−0.425** (−0.639/−0.151)	0.319 (0.028/0.560)			
MLP Rise time	0.203 (−0.096/0.468)	−0.186 (−0.455/0.113)	−0.294 (−0.540/−0.000)		
MLP Temporal anisochrony	**0.385** (0.103/0.610)	**−0.379** (−0.605/−0.097)	**−0.354** (−0.586/−0.067)	−0.084 (−0.369/0.214)	

*Critical p values are the same as in Table 1 exception made for the Bonferroni corrected p value, here 0.411. Values with a non-corrected p value < 0.01 (reasonably controlling for false positive) are reported in bold*.

### Factor analysis

The factor analysis included accuracy and speed measures in the tests measuring reading abilities, phonological awareness, temporal processing, auditory attention, and digit span. Preliminary testing showed that our model was satisfactorily adequate. Indeed the Kaiser-Meyer-Olkin (KMO) index measuring the sampling adequacy gave a value of 0.764 (recommended is >0.6). Also the Bartlett's test of sphericity rejecting the null hypothesis of an identity matrix was significant (*p* < 0.001, recommended is <0.05). Finally, following two different methods to estimate the number of factors (software package F A C T O R, Unrestricted Factor Analysis 9.2 by Urbano Lorenzo-Seva and Pere J. Ferrando) and the eigenvalue criterion ≥1, three factors were extracted explaining a variance of 61.389% (Table 4).

**Table 4:** **Varimax with Kaiser Normalization rotated factor loadings for all tests of reading, phonological awareness, temporal processing, attention and verbal short term memory, using the option “Blank” (<I0.40I)**.

	**Component**		
	**1 (6.112)**	**2 (1.644)**	**3 (1.453)**
MT text reading speed	−0.816		
Word reading accuracy	0.803		
Word reading time	0.874		
Pseudoword reading accuracy	0.813		
Pseudoword reading time	0.826		
Phonemic segmentation			0.842
Phonemic blending			0.818
Pseudoword repetition	−0.443		0.527
Auditory attention		0.671	
Digit span		0.486	
Metrical task		0.548	
Tapping		−0.586	
Rhythm reproduction		0.551	0.511
Rise time	0.540		
Temporal anisochrony		−0.802	

*The initial eigenvalues for each factor are reported in parenthesis*.

The first factor shows high factor loadings (i.e., correlation coefficients between variables and factors) for speed and accuracy scores in all reading tests and surprisingly in rise time threshold. Thus, this first factor can be interpreted as describing reading abilities.

The second factor shows high factor loadings for the temporal anisochrony threshold and auditory attention test while slightly lower factor loadings for tapping coefficient of variation, accuracy in rhythm reproduction task, musical meter perception task, pseudoword repetition test and the verbal short term memory test of WISC III. It can thus be interpreted as a factor describing broad auditory temporal processing.

The third factor shows high factor loadings for accuracy in the phonemic blending and phonemic segmentation tests and slightly lower loading for the pseudoword repetition and rhythm reproduction tasks. It can thus be interpreted as a factor describing broad phonological processing.

### Logistic regression

In the logistic regression analyses (Tables 5–8), the reading outcome measures were considered as the dependent variables.

**Table 5:** **Logistic regressions**.

**MT Text Accuracy**	**Odds ratio**	**Std. Err**.	***P* > |z|**	**95% Confidence interval**	
City	0.343	0.268	0.170	0.074	1.584
School level	1.085	0.295	0.763	0.637	1.849
IQ	0.939	0.036	0.096	0.872	1.011
Sex	0.238	0.200	0.088	0.046	1.238
Metrical task	0.641	0.124	**0.022**	0.439	0.938

*Values with a non-corrected p value < 0.01 are reported in bold*.

**Table 6:** **Logistic regressions**.

**Pseudoword reading accuracy**	**Odds ratio**	**Std. Err**.	***P* > |z|**	**95% Confidence interval**	
City	1.386	1.137	0.690	0.278	6.920
School level	1.081	0.329	0.797	0.595	1.964
IQ	0.937	0.0368	0.099	0.868	1.012
Sex	0.871	0.708	0.865	0.177	4.283
Rhythm Reproduction	0.429	0.163	**0.026**	0.203	0.903

*Values with a non-corrected p value < 0.01 are reported in bold*.

**Table 7:** **Logistic regressions**.

**Word reading accuracy**	**Odds ratio**	**Std. Err**.	***P* > |z|**	**95% Confidence interval**	
City	0.626	0.519	0.572	0.124	3.173
School level	0.658	0.189	0.146	0.375	1.156
IQ	0.968	0.039	0.418	0.895	1.047
Sex	2.050	1.773	0.407	0.376	11.170
Mother School Level	6.371	4.277	**0.006**	1.709	23.748

*Values with a non-corrected p value < 0.01 are reported in bold*.

**Table 8:** **Logistic regressions**.

**Word reading time**	**Odds ratio**	**Std. Err**.	***P* > |z|**	**95% Confidence interval**	
City	24.179	47.008	0.101	0.535	1092.288
School level	5.789	5.443	0.062	0.917	36.550
IQ	0.830	0.079	0.052	0.688	1.002
Sex	3.764	6.777	0.462	0.110	128.281
Metrical Task	0.2698	0.165	**0.032**	0.081	0.893

*Values with a non-corrected p value < 0.01 are reported in bold*.

Analyses of the MT text reading test point to the meter perception task as a good predictor of reading accuracy (*or* = 0.641, *p* = 0.02). Reading speed was only associated with the controlled variables IQ and school-level.

Analyses of the word reading test point to the mother school level as a good predictor of reading accuracy (*or* = 6.371, *p* = 0.006) and to the meter perception task as a good predictor of reading speed (*or* = 0.270, *p* = 0.032).

Analyses of the pseudoword reading test point to the rhythm reproduction test as a good predictor of reading accuracy (*or* = 0.429, *p* = 0.026). Reading speed was not significantly associated to any variables entered in the model.

## Discussion

This study explored whether and to what extent different levels of temporal processing are associated to reading and phonological abilities.

We found that rhythm reproduction were strongly associated with most reading outcome measures and phonological awareness. Furthermore, tapping tasks correlated with some aspects of language and rise time correlated with text reading, in accordance with previously published studies (Goswami et al., 2002; Thomson and Goswami, 2008).

Intriguingly, the factor analysis identified three significant factors: the first grouping reading tests and rise time thresholds; the second spanning broad auditory temporal processing, including pseudoword repetition and verbal short term memory; the third describing phonological processing but also including rhythm reproduction.

Last but not least, the logistic regression analyses indicated the meter perception task as a good predictor of text reading accuracy and word reading speed, while rhythm reproduction was the best predictor of pseudoword reading accuracy. Finally, maternal formal education level was also a good predictor of word reading accuracy.

We will first discuss the results of these complementary analyses, bridging temporal processing skills on one side and phonological awareness and literacy on the other. We will then present some considerations on the different temporal scales that are addressed by our tasks and by other tasks and models described in the literature. Finally, we will consider the use of music training as a possible rehabilitation of developmental dyslexia and give some tentative recommendations.

### Bridging temporal processing and reading skills

Correlations between the temporal processing tasks, phonology measures, and literacy confirm previously published data in the literature (Anvari et al., 2002; Overy et al., 2003; Huss et al., 2011). The temporal task showing the highest correlation is the rhythm reproduction task, followed by the tapping task. These tasks are the two most complex temporal tasks because they both require listening and motor coordination. The rhythm reproduction task also requires working memory and grouping events in meaningful chunks, even though the sequences were not long. By contrast the tapping task is a sensorimotor synchronization task which does not require working memory or chunking because the stimulus was a simple metronome.

The perceptual metrical tasks also require grouping events in chunks on the basis of a metrical hierarchy (e.g., strong-weak-weak). The independent variable was the duration of the strong beat which was sometimes lengthened by 100 ms. This is somewhat related to the two psychoacoustic tests measuring rise time and temporal anisochrony thresholds because lengthening the strong beat produces both a change in the temporal envelope of the note—like in the rise time task—and a change in the temporal relation with the preceding and following notes—like in the temporal anisochrony task. Interestingly, the temporal anisochrony task did not correlate with any phonological or literacy measures. By contrast, both the metrical and rise time tasks correlated with some literacy measures (word and text reading) pointing to a greater role of temporal envelope compared to temporal isochrony.

Results of the factor analysis confirm and extend results of the correlation matrix. Interestingly, all temporal tasks except the rise time task appear in the same factor, which also includes the auditory attention and verbal working memory (digit span) tasks. This raises the issue of the relation between attention and working memory on one side, and temporal skills on the other side. More precisely, in the case of the metrical and rhythmic reproduction tasks (but it is also the case in the text reading task), children need a global representation of the stimuli, while a serial and local representation of stimulus parts necessarily produces a poor performance. This global representation possibly needs an attentional window spanning approximately 2 s. This is also the case of the psychoacoustic task because the change to be detected was embedded in a five-note sequence for the temporal anisochrony. In the case of tapping, the temporal window is shorter when considering the interval between successive taps, but this shorter window possibly engenders a larger temporal windows, due to the emergence of a metrical structure, yielding a more global percept of several taps. In other words, when tapping along a metronome, the child will group taps together in series of two, three of four (the latter being the most likely here), with the first tap of each group being perceived as the most relevant. The third factor of the analysis shows the rhythmic task together with the phonological awareness tasks. Thus, while an attentional and memory component may indeed play a role, there seems to be a cognitive process in the rhythm reproduction task that is independent of selective attention and verbal working memory processes and that is strongly related to phonological processing. While the tapping does not appear in the third factor, this is due to the thresholding criterion we used (eigenvalue ≤ 0.4), but the tight relationship between the rhythmic task and tapping is visible in the high correlation values between these two variables.

Another interesting result of the factor analysis is the presence of the rise time task together with all reading measures. In speech, amplitude modulations in the temporal envelope (rise time) are one of the critical acoustic features underlying syllable rate and speech rhythm, and allow to distinguish between stressed and unstressed syllables (Leong et al., 2011). Indeed, amplitude modulations in the signal give a cue to the moment of occurrence of a sound that is used to build the rhythmic structure of speech (Leong and Goswami, 2014). Temporal envelope may also provide distinctive phonetic cues such as voice onset time and manner of articulation, that are necessary to discriminate otherwise similar phonemes (e.g., tie/die, bad/pad, Goswami et al., 2011a). Thus, temporal envelope is a key determinant in both perception of speech prosody and development of phonological awareness that are fundamental skills to achieve a “normal” developmental trajectory of reading (Goswami et al., 2011a). A growing body of literature attests to the presence of impaired perception of temporal envelope in developmental dyslexia, in adults and children and across languages with different phonological structures and writing systems (Goswami et al., 2011b). Interestingly, this result confirms the correlation analyses showing that this measure of rise time threshold is the only one that does not clearly correlate with the other temporal measures, exception made for a weak correlation with the meter perception task. In other words this task seems to measure a temporal scale which is not present in the other temporal tasks and which could be relevant for phonetic and prosodic processing, indispensable to all reading measures.

Correlation and factor analyses do not take into account certain sources of covariance such as age, sex, IQ and so on. However, the sources of correlation due to these variables can be controlled in regression analyses such as the logistic regression use here. In the logistic regression the dependent variables (e.g., text reading accuracy) are categorized into two categories corresponding to a severe or moderate level of dyslexia. Thus, after controlling for the effects of variables city, school-level, QI and sex, the model tests whether there is still one or more (continuous) independent variables that constitute a significant predictor of the reading outcome category. Interestingly the two measures that best predict reading outcomes are not the phonological awareness, attention or working memory tasks but the two tasks that present a greater temporal complexity, the rhythm reproduction and the metrical perceptual task. Both tasks measure a rather global level of temporal processing, including amplitude modulation, grouping events into chunks and applying a metrical hierarchy.

Although it was not the main aim of the present work, an interesting result is that mother school level was a good predictor of word reading abilities. This is probably linked to the fact that word recognition is influenced by the lexical/vocabulary development of the child (Sénéchal et al., 2006) and that maternal education is a stronger predictor of intellectual attainment than paternal education (Bradley and Corwyn, 2002). Recent research has shown the positive effect of reading during the first year of life (early literacy) on verbal competence and future academic skills (Sénéchal and LeFevre, 2002), pointing to other powerful compensatory strategies.

### Different temporal scales

One aim of the present work was to compare how different temporal skills relate to phonological and reading abilities. In doing this we had to choose a limited number of tasks, each testing a different aspect of temporal processing. We will try here to discuss how there different levels relate to each other, and how they may possibly be linked to reading disabilities in developmental dyslexia.

The smallest temporal scale is at the millisecond level. Hornickel and Kraus (2013) found that poor readers have more variable neural responses to speech; there seems to be a higher level of inconsistency in the poor reader brain's response to sound from one trial to another. Interestingly, weaker response consistency is absent with simple sounds (e.g., clicks) and present in both the formant transition (consonant) and in the more stationary part of the signal (vowel). Nonetheless, decreased consistency is maximal in the formant transition which is the most complex part of the signal. Even though the actual jitter is difficult to estimate, the lower brainstem response consistency can be accounted for by variability of the order of the millisecond or even less. While this temporal scale can be best studied by using neuroimaging techniques such as brainstem responses or cortical EEG, one should also consider that the fine-structure of speech sound (above 600 Hz) contains the formant patterns that are for instance the only acoustic cues to place of articulation (“dait” vs. “bait,” Rosen, 1992).

In her rapid auditory processing theory, Tallal (1980) proposed that the phonological deficit in developmental dyslexia could be due to impaired processing of brief, rapidly presented sounds. She proposed that children with language learning impairment (LLI) are specifically impaired in their ability to discriminate between speech sounds that are characterized by brief and rapidly successive acoustic changes. This is the case of some formant transitions characterizing the phonetic distinctive features of some consonant contrasts such as /ba/ and /da/, that can only be differentiated by the acoustic cues present within the initial 40 ms (Tallal, 2004). Tallal suggests a window of 40 ms as the critical time window of the rapid spectrotemporal acoustic changes in formant transitions that would be necessary to track temporal order across ongoing speech. Thus, the key temporal scale would be of the order of tens of milliseconds. Because recent studies have suggested a limited role for rapid auditory processing in developmental dyslexia (Heath and Hogben, 2004a,b; Thomson et al., 2013) and due to time constraints in the testing session, this time scale level was not tested in the present study, although the tapping task may draw upon temporal processing on a rapid time scale (Tiernay and Kraus, 2013b). Nonetheless, in line with the other temporal tasks that do not require speech processing and have some link with music, one possible test would be to ask children to discriminate between different musical instruments carefully manipulating the distinctive spectrotemporal features.

We have already discussed of the temporal sampling deficit framework suggested by Goswami (2011) claiming that amplitude modulations in the envelope are one of the critical acoustic properties underlying syllable rate and speech rhythm. These fluctuations range between 2 and 50 Hz, are characterized by loudness, length, attack and decay and can convey different types of linguistic information: segmental cues to manner of articulation, voicing and vowel identity. The dynamic envelope cues (changes in amplitude) can also be important suprasegmental prosodic cues to mark stresses, facilitate syllabification and normalize speech rate variations in segmental and prosodic contrasts (Rosen, 1992). In other words, whereas rapid spectro-temporal cues are thought to be linked particularly to formant transitions (Tallal, 2004), slower spectro-temporal modulations are rather linked to syllabic and prosodic structure, thus to stress patterns and speech rhythm. Already during infancy, stress patterns are important to segment, namely extract words and syllables from the speech stream, and have thus a phonological relevance (Mattys and Jusczyk, 2001), which may explain why a deficit in temporal sampling of slow amplitude modulations may deviate a normal language developmental trajectory. In the present study the measure that is more closely related to this time scale is the onset rise time threshold because it manipulates the dynamic features of amplitude envelope. However, the durational (length) and intensity (loudness) features of amplitude envelope play an important role in the metrical tasks wherein meter was marked by greater loudness of the strong beat and different trials were marked by an increased length of a strong beat note (100 ms).

Both the meter perception and rhythm reproduction tasks also require building a longer temporal structure wherein the different inter-stimuli intervals are categorized in terms of relative durations (typically simple fractions: 1/2, 1/3, 1/4 or their reciprocal) and grouped together in larger units. The temporal scale here is longer, below 2 Hz, because these larger units may contain several notes. This would correspond in speech to word segmentation (several syllables) and prosodic phrasal boundaries (several words). Moreover, these grouping phenomena give rise to the emergence of the metrical structure, the alternation of strong and weak beats which typically corresponds to the a musical bar and falls again in a rather slow temporal window (below 2 Hz). An interesting theoretical account of the perception of musical meter is given in terms of continuous attentional modulations that would be coupled via entrainment to the metrical structure of the musical stimulus (Large and Jones, 1999). In this sense, meter should not be seen as a static and quantized hierarchy of slowly alternating strong and weak beats, but as a more dynamic process that evolves in time.

The last temporal scale that we would like to address is of a somewhat different quality and not specific to the auditory domain. It concerns the ability to predict events in time. This is a more general cognitive mechanism, sometimes referred to as Bayesian inference. For instance, making a good guess by prior probabilities (i.e., our experience of the world as we know it) about which words are most likely to be heard or seen. This is especially true when the environment is “noisy” and the choice of the signal representation is ambiguous, which is the case in natural speech but also in reading (due to time pressure and competition between similar words) and even more so in children with developmental dyslexia (Norris, 2006). The use of our prior experience of the world allows predicting what event may happen and possibly when it will happen. This prior knowledge allows for a better perception of degraded speech (Sohoglu et al., 2012) as well as reading a degraded text or a text full of errors (e.g., “Aoccdrnig to a rscheearch at CmabrigdeUinervtisy”). Thus, there is intrinsic to this prediction mechanism a temporal dimension which is in this case less precisely defined, because it depends upon the context and the object to be predicted (e.g., a letter, a syllable, a word). Nonetheless, both music and speech heavily repose on this type of inference, and working on this avenue may be interesting for future research.

To conclude this section, one should keep in mind that all the different time scales that we presented above are strongly inter-related, and that the serial presentation from short to long time scale does not mean that the levels are serial or independent from each other or that embedding of one level into another only takes place in one direction.

### Music rehabilitation of developmental dyslexia

The issue raised here between the lines is whether and how music can help children with developmental dyslexia to restore a normal developmental trajectory of reading abilities. While there is not yet a clear cut answer to these questions, our data, together with other previously published results strongly suggest that music should have a positive effect on reading abilities. The reasons of this benefit are probably multiple and are still debated and will thus require further research in the years to come.

From a perspective on music and rehabilitation, it is interesting to consider the OPERA hypothesis proposed by Patel (2011), stating that music brings to adaptive brain plasticity of the same neural network involved in language processing. More precisely, this hypothesis claims that music training can drive adaptive plasticity in speech processing networks if certain conditions are respected. Firstly, a sensory or cognitive process used by both speech and music is mediated by overlapping brain networks. Secondly, music places higher demands on that process than speech. Thirdly, music engages that process with emotion, repetition, and attention (Patel, 2013).

From a more precise perspective on music and rehabilitation of developmental dyslexia, several authors have hypothesized a rehabilitation centered on rhythm, capable of developing several temporal skills that may in turn transfer to reading skills (Overy et al., 2003; Tallal and Gaab, 2006; Goswami, 2011). Nonetheless, it is not an easy issue to understand what specific aspects of temporal processing should be targeted by a possible music intervention.

Some authors suggest to work at a global level on rhythm and meter, both in perception and production (Goswami, 2011). Other researchers point to spectrotemporal processing as the best candidate to improve phonetic discrimination/categorization (Tallal and Gaab, 2006) or on both local and global dimensions, suggesting perceptual and creative games center on the musical pedagogy of Zoltan Kodaly (Overy et al., 2003).

Putting together our results with the general framework of music and language rehabilitation suggested by Patel and the more specific frameworks suggested for developmental dyslexia we will give some tentative but scientifically grounded recommendations when considering a music intervention with this population.

Our first recommendation (R1) is to use a group setting rather than an individual setting. This will possibly boost the playful and positive emotional aspects of the training and will possibly maximize rhythmic entrainment. Indeed, Kirschner and Tomasello (2009) showed that if the musical activity is realized in a social/imitative context, the synchronization ability of young children (2–3 years old) improves more compared to a context without a human partner (i.e., a computer game).

Our second recommendation (R2) is to use a fully active setting with music making and active musical games wherein music, body movements, emotions, and intentionality influence each other in a complex dynamical process (Maes et al., 2014). This will also maximize the demands on the audio-motor loop as well as on anticipatory and predictive processing, that is prediction, preparation, anticipation of events to come. In other words, music making in a social context (R1&R2) will set a high demand on Bayesian inferential efficiency, allowing for a faster prediction of future events (Bubic et al., 2010).

Our third recommendation (R3) is to focus on rhythm rather than on pitch accuracy as it is often the case in classical music pedagogy. This can be easily associated to movement and dance and, despite the idea that music has to be perfectly in tune, there are a plethora of musical games or even styles that are not too demanding on pitch accuracy, such as beat boxing, body tapping, rap and so on. This type of rhythmic activity seems to us to be the most appropriate in the rehabilitation of developmental dyslexia. On one side it will improve global temporal skills (meters and rhythm processing, sequencing, temporal prediction). On the other side, the lack or limitation of pitch and tonality will force the music teacher to make a larger use of the spectral dimension, by using different timbres produced with the mouth, body or different percussive instruments which may in turn facilitate fast temporal processing of speech sounds.

Our last recommendation (R4) is to keep variety high. While repetition is intrinsic to musical structure, the music teacher, by contrast to the computer game, can propose an almost infinite number of befittingly variations of a given game/exercise/song, that will possibly emerge in the musical interaction between the teacher and the children or the children themselves. This high variety is important in our view, to capture children attention but also to maximize the chances of a generalization process and thus a transfer to language and reading.

## Conclusions

In this study we investigated the link between different levels of temporal processing and reading skills in developmental dyslexia. We confirmed and extended previous findings describing a strong relation between timing and reading abilities. However, due to time constraints of the testing session we could not assess all temporal processing levels (for instance the fine structure level, important for phonetic discrimination). Moreover while the three statistical analyses point into a similar direction, results are only partially concordant, possibly due to the intrinsic heterogeneity of a population of dyslexic children.

Despite these limitations, our results show a strong association between reading skills and meter perception and rhythm processing. These two measures of temporal processing do not only involve timing mechanisms, but also other competences that are notoriously poor in children with developmental dyslexia, such as auditory attention (Facoetti et al., 2010) and working memory (Swanson et al., 1996). Future work should try to better tease apart the role of attention and memory in temporal processes and their link to reading skills.

The next step should be to develop interventions based on musical training for children with developmental dyslexia, and to test their efficacy through randomized controlled trials, although sufficient numerosity to allow adequate statistical power to detect treatment effects may be difficult to achieve due to the high cost and risk of drop out. A multicenter study may overcome these obstacles. To conclude, the literature review literature and our findings suggest that music training, focused on rhythm, could be beneficial for children with dyslexia, or maybe even for children identified earlier as at risk based on low phonological abilities.

### Conflict of interest statement

The authors declare that the research was conducted in the absence of any commercial or financial relationships that could be construed as a potential conflict of interest.
